# Association of Punitive and Reporting State Policies Related to Substance Use in Pregnancy With Rates of Neonatal Abstinence Syndrome

**DOI:** 10.1001/jamanetworkopen.2019.14078

**Published:** 2019-11-13

**Authors:** Laura J. Faherty, Ashley M. Kranz, Joshua Russell-Fritch, Stephen W. Patrick, Jonathan Cantor, Bradley D. Stein

**Affiliations:** 1 RAND Corporation, Boston, Massachusetts; 2 School of Medicine, Boston University, Boston, Massachusetts; 3 RAND Corporation, Arlington, Virginia; 4 RAND Corporation, Santa Monica, California; 5 Department of Pediatrics, Vanderbilt University, Nashville, Tennessee; 6 Mildred Stahlman Division of Neonatology, Vanderbilt University, Nashville, Tennessee; 7 Vanderbilt Center for Child Health Policy, Nashville, Tennessee; 8 Department of Health Policy, Vanderbilt University, Nashville, Tennessee; 9 RAND Corporation, Pittsburgh, Pennsylvania; 10 School of Medicine, University of Pittsburgh, Pittsburgh, Pennsylvania

## Abstract

**Importance:**

Despite the rapidly changing policy environment regarding substance use during pregnancy, information is lacking on the association of state policies with neonatal abstinence syndrome (NAS).

**Objective:**

To determine if punitive or reporting state policies related to substance use during pregnancy are associated with NAS rates.

**Design, Setting, and Participants:**

This repeated cross-sectional study used retrospective, difference-in-difference analysis of live births in the State Inpatient Databases from 8 US states in varying years between January 1, 2003, and December 31, 2014. States without punitive or reporting policies were compared with states with policies before and after policy enactment using logistic regression models adjusted for individual and county-level factors and state and year fixed effects. Analyses were conducted from April 10, 2019, to July 30, 2019.

**Exposures:**

Time since enactment of state policies related to substance use in pregnancy, county-level rurality and unemployment, and presence of specialized treatment programs for pregnant and postpartum women in a county.

**Main Outcome and Measures:**

Rates of NAS.

**Results:**

Among 4 567 963 live births, 23 377 neonates (0.5%) received a diagnosis of NAS. Among neonates with NAS, 3394 (14.5%) lived in counties without any treatment programs specifically for pregnant and postpartum women, 20 323 (86.9%) lived in metropolitan counties, and 8135 (34.8%) lived in counties in the highest unemployment quartile. In adjusted analyses among neonates in states with punitive policies, odds of NAS were significantly greater during the first full calendar year after enactment (adjusted odds ratio, 1.25; 95% CI, 1.06-1.46; *P* = .007) and more than 1 full year after enactment (adjusted odds ratio, 1.33; 95% CI, 1.17-1.51; *P* < .001). After regression adjustment, the annual NAS rate was 46 (95% CI, 43-48) neonates with NAS per 10 000 live births in states without punitive policies; 57 (95% CI, 48-65) neonates with NAS per 10 000 live births in states with punitive policies during the first full year after enactment; and 60 (95% CI, 56-65) neonates with NAS per 10 000 live births in states with punitive policies in effect for more than 1 full year. There was no association between reporting policies and odds of NAS.

**Conclusions and Relevance:**

In this repeated cross-sectional analysis of 8 states, states with punitive policies were associated with greater odds of NAS immediately and in the longer term, but there was no association between NAS and states with reporting policies.

## Key Points

**Question:**

Are state punitive or reporting policies related to substance use during pregnancy associated with rates of neonatal abstinence syndrome (NAS)?

**Finding:**

In this repeated cross-sectional study of nearly 4.6 million births in 8 states, policies that criminalized substance use during pregnancy, considered it grounds for civil commitment, or considered it child abuse or neglect were associated with significantly greater rates of NAS in the first full year after enactment and more than 1 full year after enactment. Policies requiring reporting of suspected prenatal substance use were not associated with rates of NAS.

**Meaning:**

Policy makers seeking to reduce NAS rates may wish to consider approaches favored by public health experts that focus on primary prevention.

## Teaser

This cross-sectional study examines the association of state-level punitive or reporting policies related to substance use during pregnancy with rates of neonatal abstinence syndrome.

## Introduction

The opioid crisis has affected a substantial number of pregnant women in the United States, with the number of pregnant women with an opioid use disorder (OUD) diagnosis at delivery quadrupling from 1999 to 2014^1^ and prenatal opioid exposure resulting in a 7-fold increase in neonatal abstinence syndrome (NAS) from 2000 to 2014.^2,3^ Neonatal intensive care unit admissions for NAS increased nearly 4-fold between 2004 and 2013,^4^ and in some hospitals, NAS accounts for approximately half of all neonatal intensive care unit days.^5^ There is substantial state-to-state variation in NAS rates,^6,7^ as well as county-level variation associated with structural factors, such as higher rates of long-term unemployment and less access to mental health care practitioners.^8^ Neonates with NAS often require prolonged and costly hospitalizations,^9^ with total hospital costs for births with NAS exceeding $500 million by 2014.^3^

Many states have long sought to address opioid misuse in pregnancy, the proximal cause of most cases of NAS, by enacting a range of policies intended to reduce opioid use in pregnancy.^10^ A number of states recently expanded policies designed to increase the availability of treatment for women with substance use disorder (SUD),^10^ adding to the number of states that had enacted such policies in the 1990s. Such policies have likely contributed to an increase in the number of SUD treatment programs specifically for pregnant and postpartum women, although such programs remain uncommon and are not easily accessible for many women.^11,12^ Many states have also responded by enacting policies that are potentially punitive toward pregnant women, such as policies considering substance use during pregnancy to be child abuse or neglect. From 2000 to 2015, the number of states with these punitive policies increased more than 2-fold from 12 to 25, and the number of states requiring health care professionals to report suspected prenatal drug abuse to child protective services or health officials increased from 12 to 23.^10^

Despite the rapidly changing policy environment, there is a paucity of information on the associations of punitive or reporting state policies being increasingly enacted since 2000 with NAS. To support more empirically informed policymaking, this study sought to address a critical gap in the literature by examining the association of states with punitive or reporting policies related to substance use during pregnancy (hereafter, *punitive* or *reporting policies*) with rates of NAS, a proxy for maternal opioid use. We hypothesized that punitive policies, which have been shown to discourage women from seeking prenatal care and SUD treatment,^13,14,15^ would be associated with higher NAS rates as women would be less likely to engage in the health care system and receive interventions that could reduce prenatal opioid use.

## Methods

The study was approved by the RAND institutional review board with a waiver of consent because data were deidentified and consent could not be feasibly obtained. This study was reported following the Strengthening the Reporting of Observational Studies in Epidemiology (STROBE) reporting guideline for observational studies.

### Data Sources

Data from the Healthcare Cost and Utilization Project’s State Inpatient Databases compiled by the Agency for Healthcare Research and Quality^16^ were used to identify rates of NAS. The State Inpatient Databases contain the universe of inpatient discharge records from community hospitals in participating states and include all patients, regardless of payer. We examined a convenience sample of states that varied in their enactment of punitive or reporting policies with the following years of data: Arkansas (2004-2009), Arizona (2003-2013), Colorado (2003-2014), Kentucky (2007-2014), Massachusetts (2005-2014), Maryland (2003-2014), Nevada (2003-2014), and Utah (2009-2014) (eTable 1 and eTable 2 in the Supplement). Data on licensed SUD treatment facilities with programs for pregnant or postpartum women were assembled from the National Directory of Drug and Alcohol Abuse Treatment Programs and geocoded using ArcGIS Desktop geographic software (Esri). This data set included treatment facilities that were licensed, certified, or otherwise approved for inclusion in the directory by their state SUD agencies and responded to the previous year’s National Survey of Substance Abuse Treatment Services. The National Survey of Substance Abuse Treatment Services include data on whether the facility has a specialized treatment program for pregnant or postpartum women. The Area Health Resources File provided additional county-level characteristics.

We examined 2 types of state policies: (1) punitive, defined as policies by which substance use during pregnancy was criminalized, considered grounds for civil commitment, or considered child abuse or neglect; and (2) reporting, defined as policies that mandated reporting of suspected prenatal substance use to relevant authorities. Information on these 2 types of policies, including effective dates, were obtained from the Guttmacher Institute,^10^ which has tracked state policies related to reproductive health and rights since the early 1970s. To obtain detailed information about state policies, such as policy enactment dates, Guttmacher Institute staff annually review the LexisNexis database, routinely monitor state legislature and state agency websites, and conduct follow-up telephone calls with policymakers as needed. We supplemented the information received from the Guttmacher Institute^10^ with information from published studies retrieved through a targeted literature review,^17^ ProPublica,^18^ and the National Conference of State Legislatures.^19^ Relevant statutes were reviewed, and we discussed discrepancies among sources and resolved them by consensus.

### Sample, Outcome, and Neonate-Level Covariates

Hospitalizations with deliveries resulting in live births (hereafter, *neonates*) were identified in the State Inpatient Databases using *International Statistical Classification of Diseases, Ninth Revision, Clinical Modification* (*ICD-9-CM*)^20^ diagnosis codes V30.00 to V30.01, V31.00 to V31.01, V32.00 to V32.01, V33.00 to V33.01, V34.00 to V34.01, V35.00 to V35.01, V36.00 to V36.01, V37.00 to V37.01, V38.00 to V38.01, or V39.00 to V39.01. Neonates were identified as having NAS if their health record included the *ICD-9-CM*^20^ diagnosis code 779.5. Consistent with prior studies,^2,3,6,7^ to exclude potentially iatrogenic cases of NAS (ie, neonates received opioids postnatally and had subsequent withdrawal), we excluded neonates with a birthweight of less than 1500 g or *ICD-9-CM* diagnosis codes 772.10 to 772.14 (intraventricular hemorrhage), 779.7 (periventricular leukomalacia), 777.50 to 777.53 (necrotizing enterocolitis), 777.6 (spontaneous intestinal perforation), 770.7 (bronchopulmonary dysplasia), or 854.0 to 854.1 (intracranial injury).^20^ Neonates were excluded if birthweight was missing or if they had *ICD-9-CM* diagnosis codes corresponding to preterm births or extreme immaturity with subclassifications for birthweight less than 1500 g (ie, 765.11 to 765.15 or 765.01 to 765.05).^20^

To control for individual factors with known associations with NAS, we identified neonate sex and created an indicator for preterm births (if *ICD-9-CM* code 765.0x or 765.1x was present).^20^ We also examined race/ethnicity as reported by the hospital (ie, non-Hispanic white, non-Hispanic black, Hispanic, or other or unknown) and expected primary payer for the birth hospitalization (ie, commercial, public coverage, or uninsured or self-pay). We excluded neonates who were missing data on sex or payer and those living in counties missing a 2003 Rural-Urban Classification Code (RUCC).^21^

### State-Level and County-Level Covariates

To examine the possibility that the association of NAS with a policy was not immediately observed, the state policy variable was categorized as no policy, the first full calendar year (hereafter, *first full year*) after the policy went into effect, and more than 1 full calendar year (hereafter, *more than 1 full year*) after the policy went into effect. Using geocoded data from the National Directory of Drug and Alcohol Abuse Treatment Programs, the presence or absence of at least 1 specialized treatment program for pregnant and postpartum women per county was identified for each year of the study sample.

Other county-level covariates from the Area Health Resources File, selected based on prior literature,^7,8,22^ included 2003 RUCC and percentage of unemployment. Missing data, which were infrequent, were imputed by taking the mean of the years before and after the missing year, or carrying forward a categorical variable unlikely to change from year to year, such as the RUCC. Consistent with other studies of the associations of urbanicity with the opioid crisis,^8,23^ counties with RUCCs of 1, 2, or 3 were classified as metropolitan; those with RUCCs of 4, 6, or 8 were considered rural adjacent; and those with RUCCs of 5, 7, or 9 were classified as rural remote. Percentage unemployment was divided into quartiles and dichotomized, with the top quartile (ie, highest unemployment) compared with the bottom 3 quartiles (ie, lower unemployment) combined.

The Area Health Resources File and the data set of treatment facilities were linked to the neonate’s county of residence using county Federal Information Processing Standards code and year. The birth hospital county was used if the neonate’s county of residence was unavailable, which occurred for births in Massachusetts (2005-2006) and Nevada (2003-2009) (8.8% of the sample).

### Statistical Analysis

Descriptive statistics are presented as frequencies and percentages of the population. The distribution of sociodemographic, clinical, and county-level characteristics were reported for neonates with and without NAS. χ^2^ tests were used to compare the distribution of variables between groups.

To examine the associations of the 2 policy types of interest with NAS, we conducted a difference-in-difference analysis to compare NAS rates before and after policy enactment in states that had a policy change using NAS rates in states without a policy change to control for secular trends. Models were adjusted for time since policy effective date and included observations for each state by year before and after policies went into effect, allowing us to compare the outcome variable in states without a policy or before a policy went into effect to states with differing lengths of time since policy enactment. Since we hypothesized that the associations of NAS with punitive policies were likely to be different from those with reporting policies, we estimated separate logit models to determine odds of NAS diagnosis for each of the policy types. Models were adjusted for neonate- and county-level characteristics, included state and year fixed effects, and clustered SEs at the state level. Using estimates from the regression models we determined the adjusted rate of NAS for neonates conditional on residing in states (1) without policies, (2) with policies during the first full year after enactment, and (3) with policies in effect for more than 1 full year, while keeping all other covariates at their original values. Two-sided *P* values were calculated, and *P* values less than .05 were considered statistically significant. Analyses were conducted using Stata statistical software version 14.2 (StataCorp) between April 10, 2019, and July 30, 2019 (eAppendix in the Supplement).

## Results

### Descriptive Results

After excluding neonates with missing data or living in counties missing data, the total sample included 4 567 963 neonates. There were 23 377 neonates (0.5%) with NAS and 4 544 586 neonates (99.5%) without NAS (Table 1). Overall, there were 51 neonates with NAS per 10 000 live births. Neonates with NAS, compared with neonates without NAS, were significantly more likely to be non-Hispanic white (16 767 neonates [71.7%] vs 2 328 723 neonates [51.2]; *P* < .001), have public coverage (19 026 neonates [81.4%] vs 1 860 850 neonates [40.9%]; *P* < .001), and be born preterm (4332 neonates [18.5%] vs 383 917 neonates [8.4%]; *P* < .001) (Table 1). Among neonates with NAS, 3394 (14.5%) lived in counties without any specialized treatment programs for pregnant and postpartum women, 20 323 (86.9%) lived in metropolitan counties, and 8135 (34.8%) lived in counties in the highest unemployment quartile, corresponding to unemployment rates of 8.1% to 29.9% (Table 1).

**Table 1.** Individual-, County-, and State-Level Characteristics of the Sample

Characteristic	Neonates, No. (%)		*P* Value
	With NAS (n = 23 377)	Without NAS (n = 4 544 586)	
**Individual Level**			
Girls	10 757 (46.0)	2 216 340 (48.8)	<.001
Race/ethnicity			
Non-Hispanic white	16 767 (71.7)	2 328 723 (51.2)	<.001
Non-Hispanic black	2017 (8.6)	464 336 (10.2)	
Hispanic	1665 (7.1)	800 758 (17.6)	
Other or unknown	2928 (12.5)	950 769 (20.9)	
Primary payer			
Public	19 026 (81.4)	1 860 850 (40.9)	<.001
Private	3159 (13.5)	2 375 452 (52.3)	
Uninsured or self-pay	1192 (5.1)	308 284 (6.8)	
Preterm delivery	4332 (18.5)	383 917 (8.4)	<.001
**County of Residence Level**^a^			
Rurality			
Metropolitan	20 323 (86.9)	4 036 258 (88.8)	<.001
Rural adjacent	1128 (4.8)	282 784 (6.2)	
Rural remote	1926 (8.2)	225 544 (5.0)	
Unemployment rate^b^			
Top quartile	8135 (34.8)	1 118 260 (24.6)	<.001
Other quartiles combined	15 242 (65.2)	3 426 326 (75.4)	
Substance use treatment programs for pregnant and postpartum women			
Present	19 983 (85.5)	3 773 161 (83.0)	<.001
Absent	3394 (14.5)	771 425 (17.0)	
**State Level**			
Punitive policy status^c^			
No policy	12 650 (54.1)	2 356 001 (51.8)	<.001
≤1 y	598 (2.6)	275 606 (6.1)	
>1 y	10 129 (43.3)	1 912 979 (42.1)	
Reporting policy status^c^			
No policy	12 520 (53.6)	2 487 109 (54.7)	<.001
≤1 y	2646 (11.3)	225 790 (5.0)	
>1 y	8211 (35.1)	1 831 687 (40.3)	
State			
Arkansas	174 (0.7)	230 133 (5.1)	<.001
Arizona	1610 (6.9)	896 506 (19.7)	
Colorado	1271 (5.4)	751 843 (16.5)	
Utah	4065 (17.4)	401 651 (8.8)	
Nevada	6702 (28.7)	730 146 (16.1)	
Kentucky	7181 (30.7)	814 370 (17.9)	
Maryland	1085 (4.6)	416 256 (9.2)	
Massachusetts	1289 (5.5)	303 681 (6.7)	

Abbreviation: NAS, neonatal abstinence syndrome.

^a^ Birth hospital county was used if neonate county of residence was unavailable (20 062 neonates [8.8%]).

^b^ Quartiles of county percentage of unemployment were divided as fourth quartile (highest), 8.1% to 29.9% unemployment; all other quartiles, 0.8% to 8.0% unemployment.

^c^ No policy represents a state-year that did not have the policy in effect or the year in which a policy went into effect; ≤1 y, the first full calendar year after the policy went into effect; and >1 y, more than 1 full calendar year after the policy went into effect.

Among neonates with NAS, 12 650 (54.1%) were born in states during years when there were no punitive policies in place; 598 (2.6%) were born in states during the first full year after punitive policy enactment; and 10 129 (43.3%) were born in states with punitive policies in effect for more than 1 full year. In addition, 12 520 neonates with NAS (53.6%) were born in states during years when there were no reporting policies in place; 2646 neonates with NAS (11.3%) were born in states during the first full year after reporting policy enactment, and 8211 neonates with NAS (35.1%) were born in states with reporting policies in effect for more than 1 full year.

### Regression Results

Adjusted odds ratios (aORs) for control variables were similar in significance, direction, and magnitude in both models, thus results are presented here for the model adjusting for state-level punitive policies. Compared with male neonates, female neonates had significantly lower odds of NAS (aOR, 0.90; 95% CI, 0.87-0.94]; *P* < .001) (Table 2). We found significantly greater odds of NAS among neonates who were publicly insured (aOR, 11.83; 95% CI, 7.67-18.24; *P* < .001), uninsured (aOR, 5.21; 95% CI, 3.92-6.9; *P* < .001), or born preterm (aOR, 2.44; 95% CI, 2.13-2.80; *P* < .001). Neonates living in counties with the highest unemployment rates had greater odds of NAS than neonates born in counties with lower unemployment rates (aOR, 1.44; 95% CI, 1.08-1.91; *P* < .001). Neonates in counties with at least 1 treatment program for pregnant and postpartum women had significantly greater odds of NAS than neonates in counties without a treatment program for this population (aOR, 1.38; 95% CI, 1.16-1.64; *P* < .001).

**Table 2.** Adjusted Models Estimating Odds of Neonatal Abstinence Syndrome Among Neonates in 8 States^a^

Covariate	Punitive Policies		Reporting Policies	
	aOR (95% CI)	*P* Value	aOR (95% CI)	*P* Value
Time since policy went into effect				
No policy^b^	1 [Reference]		1 [Reference]	
≤1 y	1.25 (1.06-1.46)	.007	1.14 (0.82-1.60)	.43
>1 y	1.33 (1.17-1.51)	<.001	1.13 (0.96-1.34)	.14
**Individual Covariates**				
Sex				
Boys	1 [Reference]	<.001	1 [Reference]	<.001
Girls	0.90 (0.87-0.94)		0.90 (0.87-0.94)	
Race/ethnicity				
Non-Hispanic white	1 [Reference]		1 [Reference]	
Non-Hispanic black	0.20 (0.16-0.26)	<.001	0.20 (0.16-0.26)	<.001
Hispanic	0.19 (0.14-0.26)	<.001	0.19 (0.14-0.26)	<.001
Other or unknown	0.37 (0.26-0.51)	<.001	0.37 (0.26-0.52)	<.001
Primary payer				
Commercially insured	1 [Reference]		1 [Reference]	
Public coverage	11.83 (7.67-18.24)	<.001	11.81 (7.65-18.22)	<.001
Uninsured or self-pay	5.21 (3.92-6.93)	<.001	5.20 (3.92-6.89)	<.001
Preterm birth	2.44 (2.13-2.80)	<.001	2.44 (2.13-2.80)	<.001
**County-Level Covariates**^c^				
Rurality				
Metropolitan	1 [Reference]		1 [Reference]	
Rural adjacent	0.68 (0.47-0.99)	.04	0.68 (0.47-0.99)	.04
Rural remote	1.22 (0.90-1.67)	.20	1.22 (0.90-1.66)	.21
Unemployment rate^d^				
Bottom 3 quartiles	1 [Reference]	.01	1 [Reference]	.01
Top quartile	1.44 (1.08-1.91)		1.46 (1.09-1.95)	
Substance use treatment programs for pregnant and postpartum women				
Absent	1 [Reference]	<.001	1 [Reference]	<.001
Present	1.38 (1.16-1.64)		1.38 (1.15-1.65)	

Abbreviation: aOR: adjusted odds ratio.

^a^ All logistic regression models are adjusted for the variables listed in the table and include state and year fixed effects with SEs clustered at the state level.

^b^ No policy either represents a state-year that did not have the policy in effect, or the year in which a policy went into effect; ≤1 y, the first full calendar year after the policy went into effect; >1 y, more than 1 full calendar year after the policy went into effect.

^c^ Birth hospital county was used if neonate county of residence was unavailable.

^d^ Quartiles of county percentage of unemployment were divided as 4th quartile (highest), 8.1% to 29.9% unemployment; all other quartiles, 0.8% to 8.0% unemployment.

Among neonates in states with punitive policies, odds of NAS were significantly greater during the first full year after enactment (aOR, 1.25; 95% CI, 1.06-1.46; *P* = .007) and more than 1 full year after enactment (aOR, 1.33; 95% CI, 1.17-1.51; *P* < .001) (Table 2). The rate of NAS per 10 000 births was significantly higher in states with punitive policies compared with states without these policies (Figure). Specifically, the annual rate of NAS was 46 (95% CI, 43-48) neonates with NAS per 10 000 live births in states without punitive policies; 57 (95% CI, 48-65) neonates with NAS per 10 000 live births during the first full year after policy enactment in states with punitive policies, an excess of 11 neonates with NAS per 10 000 live births; and 60 (95% CI, 56-65) neonates with NAS per 10 000 live births in states with punitive policies in effect for more than 1 full year, or an excess of 14 neonates with NAS per 10 000 live births (Figure). In contrast, we did not find a significant association of reporting policies with the odds of NAS at either postenactment period (Table 2).

**Figure.**
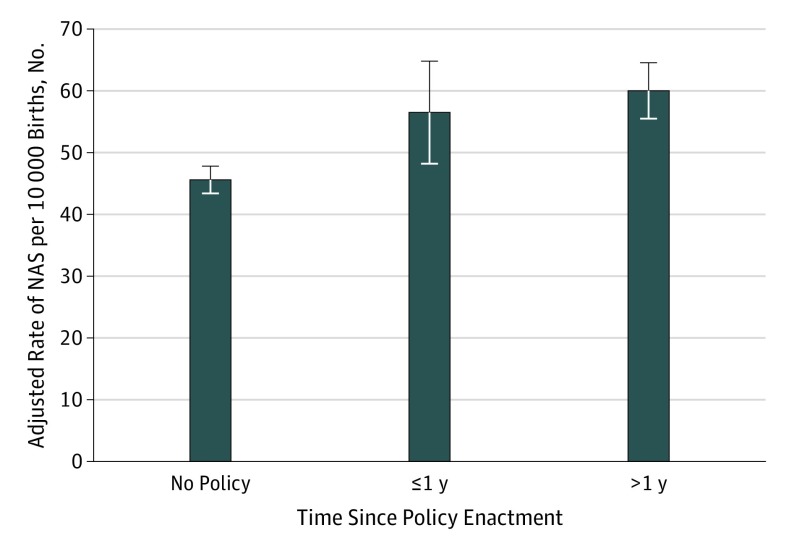
Annual Rates of Neonatal Abstinence Syndrome (NAS) per 10 000 Live Births Stratified by State Punitive Policies The adjusted rate of NAS per 10 000 live births for neonates was estimated from the regression model conditional on residing in states without punitive policies, during the first full calendar year after punitive policies went into effect, and with punitive policies in effect for more than 1 full calendar year, while keeping all other covariates at their original values. Error bars indicate 95% CI.

## Discussion

In our study of NAS rates in states that enacted punitive or reporting policies related to substance use during pregnancy, we found that the odds of NAS among neonates living in states with punitive policies were significantly greater than among neonates in states without such policies but found no association of reporting policies with odds of NAS. As policy makers react to the opioid crisis and strive to enact policies to decrease the effect of the substance use on neonates, the association of punitive policies with greater odds of NAS is noteworthy.

Punitive policies, such as considering substance use in pregnancy to be child abuse or neglect, are presumably intended to deter women from using substances during this critical period, encourage women with SUDs to seek treatment, and ensure the safety of the neonate. However, we observed greater odds of NAS in the first full year after punitive policies went into effect and beyond, suggesting that these policies were not associated with their desired outcome in either the short-term or the longer term. Our findings are consistent with prior studies showing that policies that penalized pregnant women for substance use deterred those women from seeking prenatal care and were associated with a lower prevalence of OUD diagnoses.^13,24,25,26,27^ Taken together, these studies suggest that women of reproductive age, including pregnant and postpartum women, are disengaging from the health care system in states where punitive policies regarding substance use during pregnancy have been enacted. This disengagement is hypothesized to increase NAS rates, as women in states that have enacted punitive policies may be missing key opportunities for interventions that could reduce the likelihood of having a neonate with prenatal opioid exposure (eg, avoiding or ceasing the nonmedical use of opioids, accessing family planning services, receiving mental health care to address untreated mental health comorbidity and decrease the risk of opioid misuse).^8,28,29,30^ Furthermore, among pregnant women with OUD, consistent prenatal care and SUD treatment could address 2 other risk factors for more severe and clinically apparent NAS among neonates with prenatal opioid exposure: tobacco use and use of other substances, such as benzodiazepines.^31^

In contrast, mandated reporting of substance use in pregnancy to authorities was not significantly associated with an increase or decrease in rates of NAS. We are uncertain as to why reporting policies did not have the same association as punitive policies. It may be that reporting policies were not associated with as great a disengagement from health care services as punitive policies. It is also possible that compared with punitive policies, reporting policies are more likely to result in conversations between clinicians and pregnant women that result in decreased opioid use or greater engagement in treatment for opioid related complications, actions that may decrease rates of NAS. However, qualitative studies have found that screening for suspected substance use in pregnancy, which is closely tied to reporting, can be perceived as stigmatizing and may act as a barrier to prenatal care and thus could increase NAS rates.^26,32,33^ We did not find empirical support for the belief that reporting policies are associated with lower NAS rates, presumably one of the intended results, highlighting the importance of policy makers considering strategies that enhance access to evidence-based SUD treatment for pregnant women and strengthen treatment engagement as they seek to improve outcomes for mother-neonate dyads affected by substance use in pregnancy. Several states have begun to implement state-mandated reporting of NAS for public health surveillance purposes, which can inform program planning and services, but few states take this approach.^34^ More research is needed on the association of public health reporting with NAS, as well as on how more traditional reporting policies are implemented and how they are perceived by the women most likely to be affected.

Our results on the sociodemographic factors associated with NAS are consistent with prior literature suggesting that neonates with NAS are more likely to be male, non-Hispanic white, publicly insured, and born preterm.^2,3,7,8,35^ As in prior work,^8^ we found a significant association of odds of NAS with local unemployment rates, but we did not detect an association with rurality,^7^ which may have been due to inadequate statistical power. It is notable that nearly 15% of neonates resided in counties without a treatment program specifically designed for pregnant and postpartum women and that neonates residing in counties with specialized treatment programs for pregnant and postpartum women had higher odds of NAS. We found the latter result counterintuitive and suspect it may be related to obstacles pregnant women faced accessing such programs,^11,12^ modest rates of medication treatment for pregnant women with OUD,^36,37^ and lack of coordination between such programs and prenatal care settings. Therefore, the number of pregnant women being effectively treated for OUD through such programs, despite state policies designed to expand access to SUD treatment for pregnant women,^10^ may be too small to influence NAS rates, and the significant associations we observed may have been due to these programs responding to local demand for treatment.

### Limitations

Our findings must be considered in the context of this study’s limitations. This study did not consider policy components or effectiveness of implementation, such as the frequency with which pregnant women with SUD were identified or the consequences faced, factors that may have influenced the associations of these policies with NAS. We did not consider other policies that may have been associated with maternal opioid use and engagement in treatment for SUD–such research would likely have required more state-years than were available for our analysis. Findings from a convenience sample of 8 states, selected in part to maximize our ability to examine NAS rates before and after policy enactment, while geographically diverse and with varying sociodemographic characteristics, may not be fully generalizable to the entire country or to states that have not yet enacted policy changes. Misclassification may have affected the identification of NAS, although prior research has demonstrated high positive predictive value of the *ICD-9-CM* diagnosis codes for NAS.^38^ Furthermore, NAS is a heterogeneous condition that may result from the use of medications other than opioids during pregnancy, as well as medication treatment for OUD and other substance use during pregnancy. Thus, NAS could have resulted from pregnant women receiving medication treatment, but as noted above, access to treatment, particularly pharmacotherapy, was limited for pregnant women; thus, it is unlikely to explain our results. Also, it may be that policy makers’ attention to SUD in pregnant women results in greater attention to and detection of NAS, resulting in more diagnoses. However, if that were the case, we would expect to see a comparable association of NAS rates with both punitive and reporting policies.

## Conclusions

In this cross-sectional study, our finding that punitive policies related to substance use in pregnancy were not associated with a reduction in NAS rates, and in fact, these policies may have been associated with an increase in rates of NAS, has important public health implications. Given that such policies did not appear to be associated with a decrease in rates of NAS or with enhancing the treatment of opioid-related complications in pregnant women and may in some instances have been associated with an increase in NAS rates, policy makers seeking to reduce rates of NAS may wish to consider approaches favored by public health experts that focus on primary prevention. These include responsible opioid prescribing to women of reproductive age,^25^ providing preconception and interconception physical and behavioral health care,^39^ and, given that nearly 9 in 10 pregnancies among women with OUD are unplanned,^40^ ensuring access to family planning services for women that aligns with their reproductive goals.^25,41^ Such efforts, when combined with more evidence-informed approaches to treating neonates with NAS and providing effective treatment to their mothers during the perinatal period, are likely to have the greatest effect on the prevalence and severity of NAS and on alleviating the impact of opioids on these children and their families.

## Notes

Supplement. **eTable 1.** State-Years of Data in the Sample With Year of Enactment for Punitive Policies **eTable 2.** State-Years of Data in the Sample With Year of Enactment for Reporting Policies **eAppendix.** Stata Commands Used in Difference-in-Difference Analysis of the Association of Punitive or Reporting State Policies Related to Substance Use During Pregnancy With Rates of Neonatal Abstinence Syndrome
