# Supplementary material for: Association of Punitive and Reporting State Policies Related to Substance Use in Pregnancy With Rates of Neonatal Abstinence Syndrome
Source: JAMA Netw Open. 2019 Nov 13;2(11):e1914078. doi: 10.1001/jamanetworkopen.2019.14078 (PMC6902764; doi:10.1001/jamanetworkopen.2019.14078)
Supplement: Supplement. — eTable 1. State-Years of Data in the Sample With Year of Enactment for Punitive Policies eTable 2. State-Years of Data in the Sample With Year of Enactment for Reporting Policies eAppendix. Stata Commands Used in Difference-in-Difference Analysis of the Association of Punitive or Reporting State Policies Related to Substance Use During Pregnancy With Rates of Neonatal Abstinence Syndrome [file jamanetwopen-2-e1914078-s001.pdf]

Supplementary Online Content

Faherty LJ, Kranz AM, Russell-Fritch J, Patrick SW, Cantor J, Stein BD. Association of punitive and reporting state policies related to substance use in pregnancy with rates of neonatal abstinence syndrome. *JAMA Netw Open*. 2019;2(11):e1914078. doi:10.1001/jamanetworkopen.2019.14078

eTable 1. State-Years of Data in the Sample With Year of Enactment for Punitive Policies
eTable 2. State-Years of Data in the Sample With Year of Enactment for Reporting Policies
eAppendix. Stata Commands Used in Difference-in-Difference Analysis of the Association of Punitive or Reporting State Policies Related to Substance Use During Pregnancy With Rates of Neonatal Abstinence Syndrome

This supplementary material has been provided by the authors to give readers additional information about their work.

© 2019 Faherty LJ et al. *JAMA Network Open.*

eTable 1. State-Years of Data in the Sample With Year of Enactment for Punitive Policies[a]

	Policy enacted	2003	2004	2005	2006	2007	2008	2009	2010	2011	2012	2013	2014
Arkansas	2005			**P**	*	*	*	*					
Arizona	2009							**P**	*	*	*		
Colorado	2005			**P**	*	*	*	*	*	*	*	*	*
Kentucky													
Massachusetts													
Maryland	1997	*	*	*	*	*	*	*	*	*	*	*	*
Nevada	2005			**P**	*	*	*	*	*	*	*	*	*
Utah	2012										**P**	*	*

[a] Legend: shaded boxes represent state-years of data included in the sample; P=policy enacted; *=policy present

© 2019 Faherty LJ et al. *JAMA Network Open.*

eTable 2. State-Years of Data in the Sample With Year of Enactment for Reporting Policies[a]

	Policy enacted	2003	2004	2005	2006	2007	2008	2009	2010	2011	2012	2013	2014
Arkansas													
Arizona	1998	*	*	*	*	*	*	*	*	*	*		
Colorado													
Kentucky	2013											**P**	*
Massachusetts	2008						**P**	*	*	*	*	*	*
Maryland	2013											**P**	*
Nevada	2005			**P**	*	*	*	*	*	*	*	*	*
Utah	1988							*	*	*	*	*	*

[a] Legend: shaded boxes represent state-years of data included in the sample; P=policy enacted; *=policy present

© 2019 Faherty LJ et al. *JAMA Network Open.*

eAppendix. Stata Commands Used in Difference-in-Difference Analysis of the Association of Punitive or Reporting State Policies Related to Substance Use During Pregnancy With Rates of Neonatal Abstinence Syndrome

Regression model (punitive policies)
logit NASBIRTH1 ib0.cat_yrimp_CWEL ib1.RURALITY b0.UNEMPLOYMENT_CAT b0.NUMFAC_DIC ib1.RACEi ib2.PAYERi FEMALE PRETERM i.year i.STATE, fe(year STATE)

Regression model (reporting policies)
logit NASBIRTH1 ib0.cat_yrimp_REPORT ib1.RURALITY b0.UNEMPLOYMENT_CAT b0.NUMFAC_DIC ib1.RACEi ib2.PAYERi FEMALE PRETERM i.year i.STATE, fe(year STATE)

Predictive margins (punitive policies)
margins b0.NUMFAC_DICH ib0.cat_yrimp_CWEL

Predictive margins (reporting policies)
margins b0.NUMFAC_DICH ib0.cat_yrimp_REPORT

© 2019 Faherty LJ et al. *JAMA Network Open.*
